# Mechanisms of Linezolid Resistance Among Enterococci of Clinical Origin in Spain—Detection of *optrA*- and *cfr*(D)-Carrying *E.* *faecalis*

**DOI:** 10.3390/microorganisms8081155

**Published:** 2020-07-30

**Authors:** Laura Ruiz-Ripa, Andrea T. Feßler, Dennis Hanke, Inga Eichhorn, José Manuel Azcona-Gutiérrez, Mar Olga Pérez-Moreno, Cristina Seral, Carmen Aspiroz, Carla Andrea Alonso, Luis Torres, Juan-Ignacio Alós, Stefan Schwarz, Carmen Torres

**Affiliations:** 1 Área de Bioquímica y Biología Molecular, Universidad de La Rioja, 26006 Logroño, Spain; laura_ruiz_10@hotmail.com; 2 Institute of Microbiology and Epizootics, Department of Veterinary Medicine, Freie Universität Berlin, 14163 Berlin, Germany; andrea.fessler@fu-berlin.de (A.T.F.); dennis.hanke@fu-berlin.de (D.H.); inga.eichhorn@fu-berlin.de (I.E.); stefan.schwarz@fu-berlin.de (S.S.); 3 Servicio Microbiología, Hospital San Pedro, 26006 Logroño, Spain; jmazcona@riojasalud.es (J.M.A.-G.); caalonso@riojasalud.es (C.A.A.); 4 Servicio Microbiología, Hospital Verge de la Cinta, 43500 Tortosa, Spain; marolgap@gmail.com; 5 Servicio Microbiología, Hospital Universitario Lozano Blesa, 50009 Zaragoza, Spain; cseral@salud.aragon.es; 6 Servicio Microbiología, Hospital Royo-Villanova, 50015 Zaragoza, Spain; caspirozs@gmail.com; 7 Servicio Microbiología, Hospital San Jorge, 22004 Huesca, Spain; ltorres@salud.aragon.es; 8 Servicio Microbiología, Hospital Universitario de Getafe, 28905 Getafe, Spain; nachoalos@telefonica.net

**Keywords:** whole genome sequencing, *optrA*, *cfr*(D), linezolid, *Enterococcus* spp.

## Abstract

The mechanisms of linezolid resistance among 13 *E. faecalis* and 6 *E. faecium* isolates, recovered from six Spanish hospitals during 2017–2018, were investigated. The presence of acquired linezolid resistance genes and mutations in 23S rDNA and in genes encoding for ribosomal proteins was analyzed by PCR and amplicon sequencing. Moreover, the susceptibility to 18 antimicrobial agents was investigated, and the respective molecular background was elucidated by PCR-amplicon sequencing and whole genome sequencing. The transferability of the linezolid resistance genes was evaluated by filter-mating experiments. The *optrA* gene was detected in all 13 *E. faecalis* isolates; and one *optrA*-positive isolate also carried the recently described *cfr*(D) gene. Moreover, one *E. faecalis* isolate displayed the nucleotide mutation G2576T in the 23S rDNA. This mutation was also present in all six *E. faecium* isolates. All linezolid-resistant enterococci showed a multiresistance phenotype and harbored several antimicrobial resistance genes, as well as many virulence determinants. The *fexA* gene was located upstream of the *optrA* gene in 12 of the *E. faecalis* isolates. Moreover, an *erm*(A)-like gene was located downstream of *optrA* in two isolates recovered from the same hospital. The *optrA* gene was transferable in all but one *E. faecalis* isolates, in all cases along with the *fexA* gene. The *cfr*(D) gene was not transferable. The presence of *optrA* and mutations in the 23S rDNA are the main mechanisms of linezolid resistance among *E. faecalis* and *E. faecium*, respectively. We report the first description of the *cfr*(D) gene in *E. faecalis*. The presence of the *optrA* and *cfr*(D) genes in Spanish hospitals is a public health concern.

## 1. Introduction

On the one hand, enterococci, especially the species *Enterococcus faecalis* (Efs) and *Enterococcus faecium* (Efm) can be harmless colonizers of the human intestinal tract, but on the other hand, they are also one of the most important bacterial genera related to hospital-associated infections worldwide. Enterococci possess intrinsic resistance to different antimicrobial agents commonly used to treat infections caused by Gram-positive pathogens, such as cephalosporins and aminoglycosides (low-level resistance). They also have a great capacity to acquire mobile genetic elements carrying antimicrobial resistance genes, which limits the therapeutic options [1].

Linezolid was the first oxazolidinone approved for clinical use in human medicine and is considered as a last resort antimicrobial agent. It is an important treatment option for serious infections (such as nosocomial and community-acquired pneumonia, and complicated skin and soft tissue infections) caused by multiresistant Gram-positive bacteria, including vancomycin-resistant enterococci [2,3,4].

The most common mechanism of oxazolidinone resistance among clinical isolates is attributable to point mutations in the central loop of domain V of the 23S rDNA, which, in some cases, has been related to long-term treatment with linezolid. The most common nucleotide exchange described is G2576T (*E. coli* numbering) although other mutations have been reported (e.g., C2534T, T2500A, and G2447T) [1,5]. Moreover, different amino acid exchanges, deletions, and insertions in the ribosomal proteins L3 (*rplC*), L4 (*rplD*), and L22 (*rplV*)—although of lesser significance—have also been associated with decreased susceptibility to linezolid [1,3].

To date, up to five acquired linezolid resistance genes have been described among *Enterococcus* spp.: *cfr*, *cfr*(B), *cfr*(D), *optrA*, and *poxtA*. The multiresistance gene *cfr*, which encodes an rRNA methyltransferase [6], was first described in the plasmid pSCFS1 from *Staphylococcus sciuri* [7] and was thereafter reported in various Gram-positive and Gram-negative bacteria of diverse origin [8]. Besides resistance towards oxazolidinones, it also confers resistance to phenicols, lincosamides, pleuromutilins, and streptogramin A antimicrobials (PhLOPS_A_ phenotype). In addition, variants of the *cfr* gene [*cfr*(B), *cfr*(D)] have been described in clinical enterococcal isolates [9,10,11]. However, the contribution of *cfr*-like genes to reduce the susceptibility to linezolid in enterococci is still under debate [11,12]. The *optrA* gene encodes an ATP-binding cassette F (ABC-F) protein [13] that confers resistance to oxazolidinones and phenicols. It was originally described among animal and human Efs and Efm isolates from China [14]. More recently, the new gene *poxtA*—which confers decreased susceptibility to oxazolidinones, phenicols, and tetracycline—was initially reported in a clinical methicillin-resistant *Staphylococcus aureus* strain. Like *optrA*, this gene encodes a protein of the antibiotic resistance (ARE) ABC-F family but shows only 32% amino acid identity with OptrA [15]. The *poxtA* gene has also been detected in Efs and Efm and is usually located in a composite transposon [15,16].

Although linezolid resistance remains uncommon (>99% of Gram-positive pathogens are still susceptible) [17], different antimicrobial surveillance studies/programs demonstrated that the number of linezolid-resistant *Enterococcus* (LRE) has increased during recent years [17,18,19,20]. Thus, this study aimed at determining the mechanisms of linezolid resistance and studying the molecular characteristics of LRE recovered from Spanish hospitals.

## 2. Materials and Methods

### 2.1. Bacterial Collection

A total of 13 Efs (Table 1) and six Efm isolates (Table 2) identified by matrix assisted laser desorption ionization-time of flight (MALDI-TOF) (Bruker Daltonics, Bremen, Germany) and classified as intermediate or resistant to linezolid [21], were recovered during 2017–2018 in six hospitals located in five different regions of Spain. The hospitals that took part in this study are the following: Hospital San Pedro (Logroño), Hospital Royo-Villanova (Zaragoza), Hospital Lozano Blesa (Zaragoza), Hospital Verge de la Cinta (Tortosa), Hospital San Jorge (Huesca), and Hospital Universitario de Getafe (Getafe) (Supplementary Figure S1).

### 2.2. Antimicrobial Resistance Phenotype

The susceptibility to penicillin, ampicillin, erythromycin, clindamycin, gentamicin, streptomycin, tetracycline, ciprofloxacin, levofloxacin, linezolid, vancomycin, teicoplanin, daptomycin, and sulfamethoxazole-trimethoprim was studied using the MicroScan^®^ system (Beckmann Coulter, Nyon, Switzerland). The MICs to chloramphenicol, florfenicol, kanamycin, and rifampicin were determined by broth macrodilution using *Staphylococcus aureus* ATCC^®^ 29213 and *E. faecalis* ATCC^®^ 29212 (Manassas, VA, USA) as quality controls, and to linezolid and tedizolid by E-test^®^ (bioMérieux, Durham, NC, USA). The antimicrobial susceptibility testing was performed, depending on the hospital, according to CLSI [21] or EUCAST [22] standards. The CLSI criteria [21] were used in the case of linezolid for all enterococcal isolates.

### 2.3. Mechanisms of Linezolid Resistance

All LRE were checked for the presence of the linezolid resistance genes *cfr*, *cfr*(B), *cfr*(D), *optrA*, and *poxtA* by PCR and confirmed by amplicon sequencing (Supplementary Table S1). Mutations in the domain V of the 23S rDNA were studied by PCR (Supplementary Table S1) and amplicon sequencing, and by digestion of the amplicon with the *Nhe*I restriction enzyme [23]. Mutations leading to amino acid changes in the ribosomal proteins L3, L4, and L22 were determined in all isolates by PCR and amplicon sequencing (Supplementary Table S1). The obtained sequences were compared with those of the linezolid-susceptible wild type strains *E. faecium* DO (GenBank accession number CP003583) and *E. faecalis* ATCC^®^ 29212 (GenBank accession number CP008816) using the EMBOSS Needle pairwise alignment from the EBI website.

### 2.4. Molecular Characterization

LREfs isolates were subjected to whole genome sequencing (WGS). After a pretreatment of the enterococci with an enzyme solution (20 mg/mL lysozyme, 20 mM TRIS-HCl, pH = 8, 2 mM EDTA, 1.2% Triton) for 30 min at 37 °C, 20 µL of proteinase K and 1 µL of RNAse (2 µg/µL) were added, the samples were mixed and incubated for 2 min at room temperature. Then, the DNA extraction was carried out using the QIAamp^®^ DNA Mini Kit (QIAGEN, Hilden, Germany) following the manufacturer’s instructions. The libraries for WGS were prepared using the Nextera XT library preparation kit (Illumina Inc., San Diego, CA, USA) according to the manufacturer’s instructions. The sequencing (2 × 300 bp paired-end sequencing in 40-fold multiplexes) was performed on the Illumina MiSeq (Illumina Inc., San Diego, CA, USA) platform. Sequences were *de novo* assembled using MIRA (Biomatters, Auckland, New Zealand), and annotated using RAST [24]. The nucleotide sequences were analyzed using Geneious (Biomatters, Auckland, New Zealand), and with the online tools ResFinder 3.2 [25], VirulenceFinder 2.0 [26], and MLST 2.0 [27] of the Center for Genomic Epidemiology (http://www.genomicepidemiology.org/), and ISfinder [28]. The nucleotide sequence alignments were performed using Geneious alignment with default settings and the amino acid alignments using the BLOSUM62 cost matrix. The primers used to determine the linkage of *optrA-fexA* are listed in the Supplementary Table S1.

LREfm isolates were characterized by PCR and subsequent amplicon sequencing, when applicable. On the basis of the antimicrobial resistance phenotype, the presence of the antimicrobial resistance genes *erm*(A), *erm*(B), *erm*(C), *msr*(C), *lnu*(B), *lsa*(A), *lsa*(B), *lsa*(E), *aac*(6′)-Ii, *aac*(6′)-Ie-*aph*(2″)-Ia, *aph*(3″), *ant*(6)-Ia, *str*, *tet*(K), *tet*(L), *tet*(M), *tet*(O), *fexA*, *fexB*, *cat*_pC194_, *cat*_pC221_, cat_pC223_, *catA, dfrA*, *dfrD*, *dfrG*, and *dfrK* was tested by PCR (Supplementary Table S1). PCR and amplicon sequencing were used to detect mutations that led to amino acid changes in the GyrA and ParC proteins of fluoroquinolone-resistant isolates and in the penicillin binding protein 5 (PBP5) of those isolates that were classified as resistant or intermediate to β-lactam antibiotics (Supplementary Table S1). The obtained sequences were compared with those of the reference strain *E. faecium* DO. The isolates were subjected to Multilocus Sequence Typing (MLST) following standard methodology (Supplementary Table S1). The presence of the virulence genes coding for enterococcal surface protein (*esp*), hyaluronidase (*hyl*), gelatinase (*gelE*), adhesin to collagen (*ace*) from Efs, and aggregation substances (*agg*) were checked by PCR (Supplementary Table S1).

### 2.5. Clonal Relatedness

CSI Phylogeny 1.4 [29] with default parameters, was used to map the genomes of the 13 LREfs isolates against the *E. faecalis* ATCC^®^ 29212 to infer a phylogeny-based analysis on the concatenated alignment of the high quality single nucleotide polymorphisms (SNPs). A maximum likelihood tree was constructed using FastTree 2.1.7 [29] (Lawrence Berkeley National Lab, CA, USA).

The clonal relatedness of Efm isolates was determined by pulsed-field gel electrophoresis (PFGE) of total DNA restricted with the enzyme *Sma*I, as previously described [30]. For classification of Efm as different clones, the criteria of Tenover et al. [31] were followed.

### 2.6. Conjugation Assays

Conjugation experiments were performed with all Efs isolates as donors to evaluate the transferability of the linezolid resistance genes by the filter-mating method [32]. *E. faecalis* JH2-2, which belongs to the sequence type ST8, was used as recipient strain and the selection of transconjugants was performed on BHI agar plates containing 10 mg/L florfenicol, 100 mg/L rifampicin, and 25 mg/L fusidic acid. Transconjugants were confirmed by MLST (Supplementary Table S1). The MICs of the transconjugants for different antimicrobial agents were determined by broth macrodilution using *S. aureus* ATCC^®^ 29213 and *E. faecalis* ATCC^®^ 29212 as quality controls, and by E-test^®^ [21]. The respective antimicrobial resistance genotype was investigated by PCR (Supplementary Table S1).

## 3. Results

### 3.1. Mechanisms of Oxazolidinone Resistance

The characteristics of the 13 LREfs isolates are shown in Table 1. The sources of LREfs varied but eight out of 13 isolates (62%) originated from urine samples. LREfs displayed linezolid MICs between 4–16 mg/L and all of them showed tedizolid resistance (MICs 1 mg/L). The *optrA* gene was identified in all Efs isolates (n = 13), and the X528 isolate also harbored the *cfr*(D) gene. Moreover, the nucleotide point mutation G2576T within domain V of the 23S rDNA gene was detected in one *optrA*-positive isolate. In addition, the X523 and X524 *optrA*-carrying isolates showed mutations that account for the amino acid change V109A or A114V in the ribosomal protein L4, respectively (not associated to linezolid resistance). The amino acid alignment of the deduced OptrA sequences determined in this study with the wild-type OptrA protein originally described in *E. faecalis* E349 (GenBank accession number KP399637), revealed the presence of different variants: wild-type (n = 10), Y176D and T481P (n = 2), as well as T572P (n = 1) (Table 1). The Cfr(D) of Efs X528 showed 100% amino acid identity to that of *E. faecium* 15-307-1 (GenBank accession number MG707078). Neither *cfr*, *cfr*(B), nor *poxtA* genes were detected among the LREfs.

The six LREfm isolates were recovered from surgical wounds (n = 2), blood (n = 2), urine (n = 1), and an abscess (n = 1) (Table 2). The MICs to linezolid ranged from 4 to 32 mg/L. In the LREfm isolates, the mutation G2576T in 23S rDNA was detected in all isolates (Table 2). Moreover, the C9902 and C10269 isolates showed the amino acid change A35T in the ribosomal protein L22 (not associated to linezolid resistance). The transferable linezolid resistance genes *optrA*, *poxtA*, *cfr*, *cfr*(B), and *cfr*(D) were not detected among the LREfm isolates.

### 3.2. Molecular Characterization and Clonal Relatedness

The 13 LREfs isolates belonged to the sequence types ST480 (n = 4), ST585 (n = 3), ST6 (n = 1), ST16 (n = 1), ST21 (n = 1), and ST35 (n = 1) and to the novel sequence type ST896 (n = 2) (Table 1). The Figure S2 shows the phylogenetic relatedness of the 13 Efs isolates based on the SNPs analysis. The minimum and maximum SNPs difference detected were 11 and 16567, respectively. The isolates C9737 and X526, that belonged to the novel ST896 and were recovered from the same hospital, were those that showed the minimum SNPs difference and were grouped in one phylogenetic cluster very distantly from the rest. The isolates belonging to the ST480 and ST585 were grouped in two phylogenetic clusters in accordance with their STs (Figure S2).

In the case of the LREfm, four out of six isolates were assigned to ST117, and the others to ST17 or ST262 (Table 2). The PFGE fragment patterns revealed that all six isolates belonged to different clones (A to F) (Figure S3).

### 3.3. Resistance to Non-Oxazolidinone Agents

All LRE showed a multidrug resistance phenotype (resistant to three or more classes of antimicrobial agents), but were susceptible to vancomycin, teicoplanin and daptomycin, among others (Table 1 and Table 2). All LREfm showed penicillin and ampicillin resistance and exhibited several amino acid changes in the deduced sequence of the PBP5 (Table 2). All LRE displayed resistance to macrolides and lincosamides with the detection of different combinations of the resistance genes *erm*(A), *erm*(B), *msr*(C), *lnu*(B), *lsa*(A), and *lsa*(E). High-level resistance to at least one aminoglycoside was detected in 12 LREfs and in all LREfm, mediated by the resistance genes *aac*(6′)-*aph*(2″), *aac*(6′)-Ii, *aph*(3′)-III, *ant*(6)-Ia, and/or *str*. Tetracycline resistance was found in all LREfs isolates and in one LREfm isolate mediated by *tet*(L), *tet*(M), and/or *tet*(O) genes. The analysis of the WGS and the PCR-amplicon sequencing results revealed the presence of amino acid changes in the GyrA (S83Y or S83I) and/or ParC (S80I) proteins in fluoroquinolone-resistant isolates (11 LREfs and all LREfm). All LREfs isolates showed resistance to florfenicol (MICs 32–64 mg/L) and chloramphenicol (MICs 32–128 mg/L) and harbored the *fexA* gene, but were negative for the *fexB* gene. The *catA* and *cat*_pC221_ genes were also detected among LREfs. All LREfm, in contrast, showed susceptibility to chloramphenicol and florfenicol. Eight LREfs and all LREfm isolates harbored the trimethoprim resistance gene *dfrG* and these isolates were also classified as resistant to sulfamethoxazole-trimethoprim (Table 1 and Table 2).

### 3.4. Virulence Profile

In total, 22 different virulence genes were detected among the LRE isolates, including those implicated in biofilm formation, bacterial adherence, and production of cytolysins, among others (Table 1 and Table 2). Ten of these virulence genes (*ace*, *efaAfs*, *elrA*, *srtA*, *ccf*, *cob*, *cad*, *camE*, *ebpA*, and *tpx*) were detected in all LREfs. The gene for the *Enterococcus* surface protein, *esp*, was present in one LREfs and four LREfm isolates. One LREfm isolate did not contain any of the virulence genes studied (Table 1 and Table 2).

### 3.5. Genetic Environment of the optrA and cfr(D) Genes

**Figure 1:**
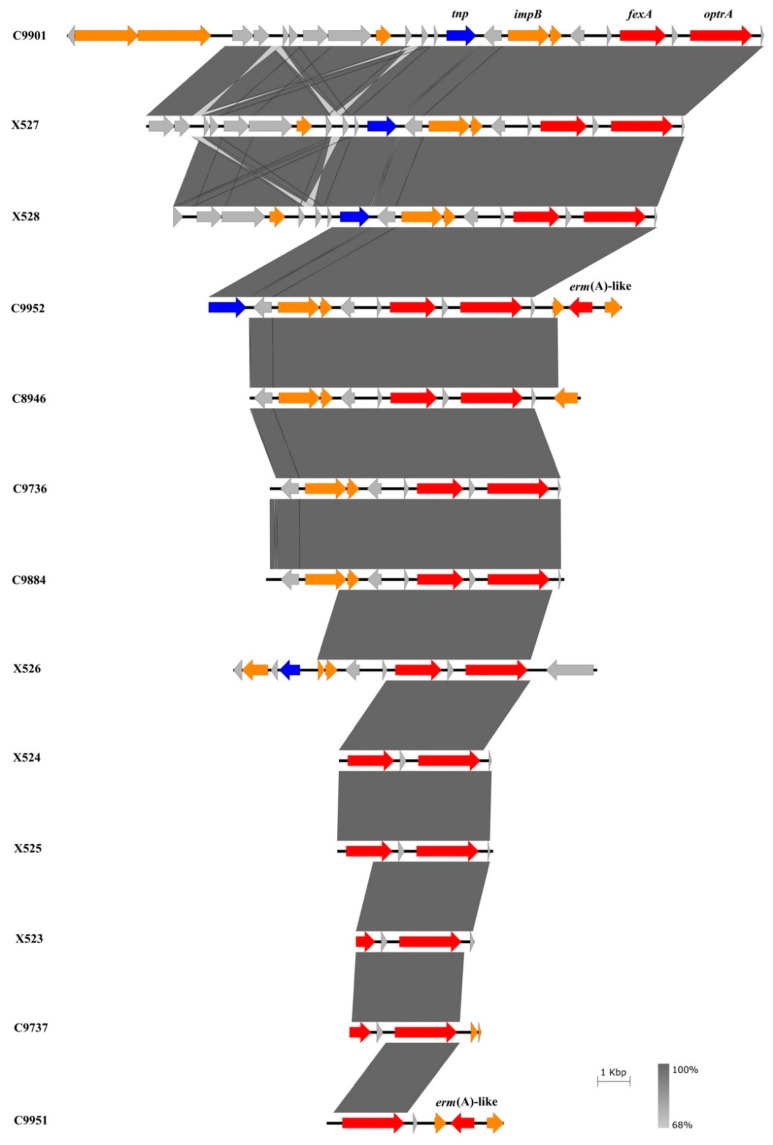
BLAST comparison of the genetic environment of the *optrA* gene in the 13 clinical LREfs isolates. Coding sequences (CDS) are colored according to their function: red, antimicrobial resistance; blue, recombination and transposition; orange, known function; grey, unknown function. The percentage of identity and scale bar legends are shown on the right.

The nucleotide sequence alignment of the genetic environment of the linezolid resistance gene *optrA* in the 13 LREfs isolates is shown in Figure 1. The genetic context showed different degrees of nucleotide sequence identity with one another although in some isolates the *optrA* flanking sequences could only be partially identified due to the contig length. The region upstream of the *optrA* gene showed high sequence similarity in the isolates C9901 (GenBank accession number MN848142), X527, X528, C9952 (GenBank accession number MN731744), C8946, C9736, and C9884 although they were recovered from different hospitals. In the isolates C9901, X527, X528, and C9952, the *tnp* gene encoding an IS*L3* family transposase and the *impB* gene which is involved in DNA repair, were detected upstream of the *optrA* gene. In the case of isolate X526 (GenBank accession number MN731743), one site-specific recombinase gene was identified. The *fexA* gene, which confers resistance to fluorinated and non-fluorinated phenicols, was detected upstream of the *optrA* gene in 12 isolates, although in the isolates X523 and C9737 it could not be totally revealed because of incomplete sequencing with the primers used. Moreover, an *erm*(A)-like gene was located downstream of the *optrA* gene in two isolates recovered from the same hospital. This *erm*(A)-like gene showed 100% nucleotide identity to that detected in the *optrA* gene cluster of plasmid p10-2-2 of *E. faecalis* strain 10-2-2 (GenBank accession number KT862775) [33]. We did not identify any *rep* gene or insertion sequence elements (ISs) in the genetic context of the *optrA* gene in the 13 LREfs investigated.

Regarding the *cfr*(D) gene, we could identify the presence of a *guaA* gene encoding a glutamine-hydrolyzing guanosine monophosphate synthase in the downstream region. Upstream of the *cfr*(D) gene, due to the short sequence length, we could only detect three reading frames for proteins with unknown function. The 4545 bp of the genetic environment of the *cfr*(D) gene revealed 100% nucleotide identity to that of plasmid 4 of *E. faecium* E8014 (GenBank accession number LR135354).

### 3.6. Transferability of the optrA and cfr(D) Genes

The *optrA* gene was successfully transferred by conjugation to *E. faecalis* JH2-2 in 12 out of the 13 isolates. The single isolate for which the *optrA* gene was not transferred was the isolate C8946. The *optrA* gene was transferred in all cases together with the *fexA* gene (Table 3). All transconjugants (TC) exhibited resistance to chloramphenicol (MICs 32–64 mg/L), florfenicol (MICs 32–64 mg/L), tedizolid (MICs 1 mg/L), and were classified as intermediate or resistant to linezolid (MICs 4–8 mg/L). Moreover, the transconjugants TC-C9951 and TC-C9952 showed resistance to erythromycin (MICs 128–256 mg/L) and clindamycin (MICs >256 mg/L) and harbored the *erm*(A)-like gene. In addition, TC-9951 was resistant to tetracycline and carried the resistance genes *tet*(L) and *tet*(O) (MIC 128 mg/L) (Table 3). The *cfr*(D) gene was not transferable.

## 4. Discussion

Since linezolid is a last resort antimicrobial agent in the treatment of serious infections caused by Gram-positive pathogens, linezolid resistance represents a great public health concern. Hence, it is very important to determine the mechanisms of this resistance in clinical isolates. The first description of LRE in Spain occurred in 2003, shortly after the introduction of this antimicrobial agent for clinical use in humans in 2001 in this country [34]. Since then, the occurrence of linezolid resistance among enterococci has increased considerably over recent years in Spain and worldwide [3,4,14,17,18,19,20,35,36]. Spreading linezolid resistance is not only a problem in the clinical setting but it has also become a concern in the livestock environment caused by the presence of transferable linezolid resistance genes [1,4,14,33]. Traditionally, mutations in domain V of 23S rDNA have been considered as the main mechanism involved in linezolid resistance among clinical isolates [1,3,4,5]. However, this study and other previous findings have demonstrated changes in the mechanisms of linezolid resistance in the clinical setting [14,18,19,20,36,37,38,39,40,41,42].

In this work, linezolid resistance was detected in the species *E. faecalis* and *E. faecium.* This could be expected since they are the most common enterococcal species related to hospital-associated infections [1,4,5]. However, in the clinical setting, previous studies reported the presence of linezolid resistance in other enterococcal species such as *E. thailandicus*, *E. gallinarum*, and *E. avium* [31,34]. The *optrA* and *cfr*(D) genes were the only linezolid resistance genes detected in our study, although the *cfr*, *cfr*(B), and *poxtA* genes have been previously detected in Efs and/or Efm of clinical origin [10,16,41]. In Spain, a recent study conducted among clinical LRE of different hospitals revealed a high prevalence of the *optrA* gene among LREfs isolates and the presence of two *poxtA*-carrying isolates, while *cfr*-like genes were absent [37].

The *optrA* gene is widely spread among enterococci in different countries, but especially present in China [14,18,19,38,39,40,41,42]. In the case of Spain, more than the 80% of LREfs isolates recovered from Spanish hospitals during 2015–2018 carried the *optrA* gene [37]. Generally, the *optrA* gene confers relatively low MICs to linezolid (4–16 mg/L) [14,17,18,19,35,36,38,39,40,41,42], which is in agreement with our results. So far, numerous variants of the *optrA* gene have been described in Efs and Efm from humans, animals, and food items and some of these variants seemed to be associated with higher or lower linezolid MICs [43,44]. In this study, the amino acid changes Y176D and T481P in OptrA, DP (aspartic acid-proline) variant, were detected in two Efs isolates. This variant has already been described in clinical isolates from China [18,38,43,44]. However, one isolate showed a novel amino acid change in OptrA at position T572P.

In this study, we report, to the best of our knowledge, the first description of the *cfr*(D) gene in the species *E. faecalis*. The Cfr(D) shares 64% amino acid identity to Cfr, but there is controversy about its contribution to linezolid resistance, as it confers a PhLOPS_A_ resistance phenotype when expressed in *Escherichia coli* but not in Efs or Efm [11]. The *cfr*(D) gene has only been detected in clinical Efm isolates from France and Australia, and, as the isolate of this study, they co-harbored the *optrA* gene [11,45].

The G2576T mutation in the 23S rDNA is the main mechanism implicated in linezolid resistance of Efm isolates investigated in this study, which is in accordance with previous findings [3,19,35,37]. A low rate of amino acid changes in the ribosomal proteins has been observed among LRE in this study, which is consistent with previous studies which observed that these are more commonly found among clinical coagulase-negative staphylococci than in *S. aureus* or enterococci [3]. As far as we know, the amino acid changes detected in the ribosomal proteins L4 (V109A and A114V) and L22 (A35T) in this study have not been reported in LRE. Different amino acid changes in these ribosomal proteins have been previously described in linezolid-susceptible strains [3,42], so it is questionable whether the mutations detected in this study are associated with decreased susceptibility to linezolid; for this reason, these amino acid changes have not been included in Table 1 and Table 2.

In this study, LREfs isolates belonged to seven different STs, but the predominance (eight out of 13 isolates) of isolates typed as ST480, ST585, or ST16 is in accordance with prior studies among clinical *optrA*-positive LREfs in Spain [37] and worldwide [14,20,40]. However, we did not detect the ST116, which is also frequently found among *optrA*-positive Efs of different origins [14,40]. Regarding Efm, all isolates were assigned to the hospital-associated subclade A1, which is the most important genogroup among Efm related to clinical infections and responsible for hospital outbreaks worldwide [46]. Efm isolates belonging to this subclade recovered from both humans and livestock have been previously reported as carrying the *optrA* gene [14,37].

The multiresistance phenotypes and genotypes observed are commonly seen among LRE [35,40]. Fortunately, all isolates in our study were susceptible to daptomycin and vancomycin, which would be considered as alternative antimicrobial options. The resistance rates to macrolides and lincosamides (100%), as well as fluoroquinolones (89%), detected in this study were very high, which is in accordance with previous studies among clinical LRE [35,40]. As reported by other authors for clinical LRE [30,36], the examined isolates harbored various virulence genes of great importance for the pathogenesis of enterococci. Many of these virulence determinants, such as the collagen binding protein Ace and the pilus protein EbpA, are shared by all isolates and seem to be generally widespread among LREfs of clinical origin [36,40]. The *esp* gene is one of the most important enterococcal virulence genes, which contributes to the colonization and persistence of enterococcal infections and is frequently detected among isolates recovered from human infections. Moreover, four out of six LREfm isolates carried the virulence gene *esp*, which is known to be located on a putative pathogenicity island that is present in the majority of Efm isolates belonging to the subclade A1 [46].

The *optrA* gene was confirmed to have a great capacity to disseminate among different Gram-positive bacteria [14]. It is often located on conjugative plasmids that commonly carry other antimicrobial resistance genes and is frequently found in combination with the *fexA* gene [14,33,37,44]. Likewise, when *optrA* is located in the chromosomal DNA, the *fexA* gene is often identified in its genetic environment [20,23,37,44]. In 12 out of 13 LREfs isolates of our study, the *fexA* gene was located upstream of *optrA*. The remaining Efs isolate also carried the *fexA* gene but the sequenced genetic environment of *optrA* did not cover the possible presence or absence of *fexA*. When located on plasmids, the insertion sequence IS*1216E* was frequently identified upstream and/or downstream of the *optrA* gene [33,36,37,44], however, we did not detect this IS*1216E* element. Moreover, we also did not detect the putative transcriptional regulator *araC* commonly located just upstream the *optrA* gene [33,43,44] when located in the chromosomal DNA. The *impB* and *tnp* genes, both detected in four LREfs isolates, are commonly found as part of the *optrA* gene cluster of different plasmids, such as in *E. faecalis* strain XY17 plasmid pXY17 (GenBank accession number KT862780) [33]. The antimicrobial susceptibility of the transconjugants is in accordance with the resistance genotype detected. The transferability of the *optrA* gene together with other antimicrobial resistance genes in 12 LREfs isolates suggests its possible plasmid location; however, additional experiments would be required to confirm this point. Besides *optrA*, the *erm*(A)-like gene was detected in the transconjugants TC-9951 and TC-9952, which was expected, since it was located downstream of *optrA* in both isolates. The *tet*(L) and *tet*(O) genes were transferred together with *optrA*, *fexA*, and the *erm*(A)-like gene in the case of TC-C9951. These tetracycline resistance genes were detected on the same contig, which was identical—as well as the contigs containing *optrA*, *fexA*, and *erm*(A) genes—to the respective parts of *Enterococcaceae* strain E508 plasmid pE508 (GenBank accession number MK425645). This observation suggests that these five resistance genes could be located on the same plasmid.

Although the co-transferability of the *optrA* and *cfr*(D) genes by conjugation has been previously described in the species *E. faecium* [11], in this work the *cfr*(D) gene was not transferable.

## 5. Conclusions

In conclusion, we have shown that the main mechanism involved in linezolid resistance among clinical LREfs is conferred by the *optrA* gene, whereas in LREfm the presence of mutations in the domain V of the 23S rDNA is the major cause for linezolid resistance. Moreover, we report the first detection of the *cfr*(D) gene in Efs. Some of the most important virulence factors of *Enterococcus* spp. were detected among LRE, which, if coupled with the multidrug resistance phenotype, is a public health concern.

## Figures and Tables

**Table 1:** Characterization of 13 clinical linezolid-resistant *E. faecalis* isolates investigated in this study.

Isolate	Hospital 1	Year	Type of Sample	MLST/CC	Linezolid MIC (mg/L)	Mechanisms of Linezolid Resistance 2	Antimicrobial Resistance Phenotype 3	Antimicrobial Resistance Genotype	Virulence Genes
C9884	HSJ	2017	Urine	ST585/CC4	16	*optrA*	ERY-CLI-GEN-KAN-STR-TET-CHL-FFC-LZD-TDZ-SXT-CIP ^4^-LEV	*erm*(B), *lnu*(B), *lsa*(A), *lsa*(E), *aac*(6′)-Ie-*aph*(2″)-Ia, *aph*(3″)-III, *ant*(6)-Ia, *str*, *tet*(M), *tet*(L), *optrA*, *fexA*, *dfrG*	*gelE*, *ace*, *agg*, *efaAfs*, *elrA*, *srtA*, *ccf*, *cob*, *cad*, *camE*, *ebpA*, *ebpC*, *fsrB*, *tpx*
C8946	HUG	2017	Leg wound exudate	ST480	16	*optrA*	ERY-CLI-GEN-KAN-STR-TET-CHL-FFC-LZD-TDZ-SXT-CIP ^4,5^-LEV	*erm*(B), *lnu*(B), *lsa*(A), *lsa*(E), *aac*(6′)-Ie-*aph*(2″)-Ia, *aph*(3″)-III, *ant*(6)-Ia, *tet*(M), *tet*(L), *optrA*, *fexA*, *dfrG*	*hylA*, *ace*, *efaAfs*, *elrA*, *srtA*, *ccf*, *cob*, *cad*, *camE*, *ebpA*, *ebpB*, *tpx*
C9901	HUG	2017	Urine	ST21/CC21	16	*optrA*	ERY-CLI-KAN-STR-TET-CHL-FFC-LZD-TDZ	*erm*(B), *lnu*(B), *lsa*(A), *lsa*(E), *aph*(3″)-III, *ant*(6)-Ia, *tet*(M), *tet*(L), *optrA*, *fexA*	*gelE*, *hylA*, *hylB*, *ace*, *agg*, *cylL*, *efaAfs*, *elrA*, *srtA*, *ccf*, *cob*, *cad*, *camE*, *ebpA*, *ebpC*, *fsrB*, *tpx*
C9951	HSP	2017	Urine	ST16	8	*optrA* (Y176D, T481P); 23S rDNA (G2576T)	ERY-CLI-GEN-KAN-STR-TET-CHL-FFC-LZD-TDZ-SXT	*erm*(A)-like, *erm*(B), *lnu*(B), *lsa*(A), *lsa*(E), *aac*(6′)-Ie-*aph*(2″)-Ia, *aph*(3″)-III, *ant*(6)-Ia, *tet*(M), *tet*(L), *tet*(O), *optrA*, *fexA*, *dfrG*	*ace*, *agg*, *cylA*, *efaAfs*, *elrA*, *srtA*, *ccf*, *cob*, *cad*, *camE*, *ebpA*, *ebpC*, *tpx*
C9952	HSP	2017	Urine	ST35	8	*optrA* (Y176D, T481P)	ERY-CLI-GEN-KAN-STR-TET CHL-FFC-LZD-TDZ-CIP ^4,6^-LEV	*erm*(A)-like, *erm*(B), *lnu*(B), *lsa*(A), *lsa*(E), *aac*(6′)-Ie-*aph*(2″)-Ia, *aph*(3″)-III, *ant*(6)-Ia, *tet*(M), *tet*(L), *optrA*, *fexA*	*gelE*, *hylB*, *ace*, *cylA*, *cylL*, *cylM*, *efaAfs*, *elrA*, *srtA*, *ccf*, *cob*, *cad*, *camE*, *ebpA*, *ebpB*, *fsrB*, *tpx*
C9736	HVC	2017	Urine	ST585/CC4	16	*optrA*	ERY-CLI-GEN-KAN-STR-TET-CHL-FFC-LZD-TDZ-SXT-CIP ^4^-LEV	*erm*(B), *lnu*(B), *lsa*(A), *lsa*(E), *aac*(6′)-Ie-*aph*(2″)-Ia, *aph*(3″)-III, *ant*(6)-Ia, *str*, *tet*(M), *tet*(L), *optrA*, *fexA*, *dfrG*	*gelE*, *ace*, *agg*, *cylL*, *efaAfs*, *elrA*, *srtA*, *ccf*, *cob*, *cad*, *camE*, *ebpA*, *ebpC*, *fsrB*, *tpx*
C9737	HVC	2017	Urine	ST896	16	*optrA*	ERY-CLI-KAN-STR-TET-CHL-FFC-LZD-TDZ-CIP ^4,5^-LEV	*erm*(B), *lnu*(B), *lsa*(A), *lsa*(E), *aph*(3″)-III, *ant*(6)-Ia, *tet*(M), *optrA*, *fexA*, *cat*_pC221_	*esp*, *gelE*, *hylA*, *hylB*, *ace*, *agg*, *efaAfs*, *elrA*, *srtA*, *ccf*, *cob*, *cad*, *camE*, *ebpA*, *ebpB*, *tpx*
X523	HVC	2018	Otic exudate	ST480	8	*optrA* (T572P)	ERY-CLI-TET-CHL-FFC-LZD-TDZ-SXT-CIP ^4,5^-LEV	*lsa*(A), *lsa*(E), *tet*(M), *tet*(L), *optrA*, *fexA, dfrG*	*hylA*, *ace*, *efaAfs*, *elrA*, *srtA*, *ccf*, *cob*, *cad*, *camE*, *ebpA*, *ebpB*, *tpx*
X524	HVC	2018	Urine	ST480	8	*optrA*	ERY-CLI-GEN-KAN-STR-TET-CHL-FFC-LZD-TDZ-SXT-CIP ^4,5^-LEV	*erm*(B), *lsa*(A), *lsa*(E), *aac*(6′)-Ie-*aph*(2″)-Ia, *aph*(3″)-III, *ant*(6)-Ia, *tet*(M), *tet*(L), *optrA*, *fexA*, *dfrG*	*hylA*, *ace*, *agg*, *cylL*, *cylM*, *efaAfs*, *elrA*, *srtA*, *ccf*, *cob*, *cad*, *camE*, *ebpA*, *ebpB*, *tpx*
X525	HVC	2018	Ulcer	ST585/CC4	8	*optrA*	ERY-CLI-GEN-KAN-STR-TET-CHL-FFC-LZD-TDZ-SXT-CIP ^4^-LEV	*erm*(B), *lnu*(B), *lsa*(A), *lsa*(E), *aac*(6′)-Ie-*aph*(2″)-Ia, *aph*(3″)-III, *ant*(6)-Ia, *str*, *tet*(M), *tet*(L), *optrA*, *fexA*, *catA*, *dfrG*	*gelE*, *ace*, *agg*, *cylL*, *efaAfs*, *elrA*, *srtA*, *cob*, *cad*, *camE*, *ebpA*, *ebpC*, *fsrB*, *tpx*
X526	HVC	2018	Urine	ST896	4	*optrA*	ERY-CLI-KAN-STR-TET-CHL-FFC-LZD-TDZ-CIP ^4,5^-LEV	*erm*(B), *lnu*(B), *lsa*(A), *lsa*(E), *aph*(3″)-III, *ant*(6)-Ia, *tet*(M), *optrA*, *fexA*, *cat*_pC221_	*gelE*, *hylA*, *hylB*, *ace*, *cylA*, *cylL*, *efaAfs*, *elrA*, *srtA*, *ccf*, *cob*, *cad*, *camE*, *ebpA*, *ebpB*, *fsrB*, *tpx*
X527	HVC	2018	Ulcer	ST6/CC2	16	*optrA*	ERY-CLI-GEN-KAN-STR-TET-CHL-FFC-LZD-TDZ-CIP ^4,5^-LEV	*erm*(B), *lsa*(A), *aac*(6′)-*aph*(2″), *aph*(3″)-III, *ant*(6)-Ia, *tet*(M), *optrA*, *fexA*	*gelE*, *hylA*, *hylB*, *ace*, *agg*, *efaAfs*, *elrA*, *srtA*, *cob*, *cad*, *camE*, *ebpA*, *ebpB*, *ebpC*, *fsrB*, *tpx*
X528	HVC	2018	Abscess	ST480	16	*optrA; cfr*(D)	ERY-CLI-KAN-STR-TET-CHL-FFC-LZD-TDZ-SXT-CIP ^4,5^-LEV	*erm*(B)*, lsa*(A), *aph*(3′’)-III, *ant*(6)-Ia*, tet*(M), *tet*(L), *cfr*(D), *optrA*, *fexA, dfrG*	*hylA*, *ace*, *agg*, *efaAfs*, *elrA*, *srtA*, *ccf*, *cob*, *cad*, *camE*, *ebpA*, *ebpB*, *tpx*

^1^ HSJ, Hospital San Jorge; HUG, Hospital Universitario de Getafe; HSP, Hospital San Pedro; HVC, Hospital Verge de la Cinta; ^2^ In brackets the mutations or amino acid changes detected;^3^ ERY, erythromycin; CLI, clindamycin; GEN, gentamicin; KAN, kanamycin; STR, streptomycin; TET, tetracycline; CIP, ciprofloxacin; LEV, levofloxacin; CHL, chloramphenicol; FFC, florfenicol; LZD, linezolid; TDZ, tedizolid; SXT, sulfamethoxazole-trimethoprim-; ^4^ Amino acid change S80I in the ParC protein; ^5^ Amino acid change S83Y in the GyrA protein; ^6^ Amino acid change S83I in the GyrA protein.

**Table 2:** Characterization of the six clinical linezolid-resistant *E. faecium* isolates investigated in this study.

Isolate	Hospital 1	Year	Type of Sample	PFGE Pattern	MLST	LZD MIC (mg/L)	Mechanisms of LZD Resistance 2	Antimicrobial Resistance Phenotype 3	Antimicrobial Resistance Genotype 4	Virulence Genes
C9902	HLB	2017	Abscess	A	ST17	8	23S rDNA (G2576T)	PEN ^5^-AMP-ERY-CLI-KAN-STR-CIP ^8^-LEV-LZD-SXT	*erm*(B), *msrC*, *aac*(6′)-Ii, *aph*(3″)-III, *ant*(6)-Ia, *dfrG*	*esp, hyl*
C9903	HLB	2017	Surgical wound	B	ST262	16	23S rDNA (G2576T)	PEN ^5^-AMP-ERY-CLI-GEN-KAN-STR-TET-CIP ^8^-LEV-LZD-SXT	*ms*r(C), *lsa*(E), *aac*(6′)-Ie-*aph*(2″)-Ia, *aac*(6′)-Ii, *ant*(6)-Ia, *tet*(M), *tet*(L), *dfrG*	
C10262	HLB	2017	Blood	C	ST117	32	23S rDNA (G2576T)	PEN ^6^-AMP-ERY-CLI-KAN-STR-CIP ^8^-LEV-LZD-SXT	*erm*(B), *ms*r(C), *aac*(6′)-Ii, *aph*(3″)-III*, ant*(6)-Ia, *dfrG*	*esp, hyl*
C9950	HSP	2017	Blood	D	ST117	4	23S rDNA (G2576T)	PEN ^7^-AMP-ERY-CLI-KAN-STR-CIP ^8^-LEV-LZD-SXT	*erm*(B), *ms*r(C), *aac*(6′)-Ii, *aph*(3″)-III, *ant*(6)-Ia, *dfrG*	*esp*, *hyl*
C9953	HSP	2017	Urine	E	ST117	16	23S rDNA (G2576T)	PEN ^I,5^-AMP-ERY-CLI-KAN-STR-CIP ^8^-LEV-LZD-SXT	*erm*(B), *ms*r(C), *aac*(6′)-Ii, *aph*(3″)-III, *ant*(6)-Ia, *dfrG*	*hyl*
C10269	HRV	2017	Surgical wound	F	ST117	16	23S rDNA (G2576T)	PEN ^6^-AMP-ERY-CLI-KAN-STR-CIP ^8^-LEV-LZD-SXT	*erm*(B), *ms*r(C), *aac*(6′)-Ii, *aph*(3″)-III, *ant*(6)-Ia, *dfrG*	*esp, hyl*

^1^ HLB, Hospital Lozano Blesa; HSP, Hospital San Pedro; HRV, Hospital Royo Villanova;^2^ In brackets the mutations detected; ^3^ PEN, penicillin; AMP, ampicillin; ERY, erythromycin; CLI, clindamycin; GEN, gentamicin; KAN, kanamycin; STR, streptomycin; TET, tetracycline; CIP, ciprofloxacin; LEV, levofloxacin; LZD, linezolid; SXT, trimethoprim-sulfamethoxazole. I, intermediate; ^4^ The *msrC* and *aac*(6′)-Ii resistance genes are intrinsic to *E. faecium;* ^5^ Amino acid changes detected in the penicillin binding protein 5 (PBP5): G66E, A68T, E85D, E100Q, K144Q, T172A, L177I, D204G, A216S, T324A, V462A, insertion 466S, M485A, N496K, A499T, E525D, N546T, A558T, G582S, E629V, K632Q, P642L, D644N, and P667S; ^6^ Amino acid changes detected in the PBP5: G66E, A68T, E85D, E100Q, K144Q, T172A, L177I, D204G, A216S, T324A, insertion 466S, M485A, N496K, F497I, A499T, E525D, N546T, A558T, G582S, E629V, K632Q, P642L, D644N, and P667S; ^7^ Amino acid changes detected in the PBP5: G66E, E85D, E100Q, K144Q, T172A, L177I, D204G, A216S, T324A, V462A, insertion 466S, M485A, N496K, A499T, E525D, N546T, A558T, G582S, E629V, K632Q, P642L, D644N, and P667S; ^8^ Amino acid change S80I and S83Y in the ParC and the GyrA proteins, respectively.

**Table 3:** Minimum inhibitory concentrations (MICs) of the recipient *E. faecalis* JH2-2, the *E. faecalis* donor strains, and the respective transconjugants.

Strain	Genotype	MIC (mg/L) ^1^						
		ERY	CLI	TET	CHL	FFC	LZD	TDZ
***E. faecalis*** **JH2-2 (recipient)**	-	1	2	0.125	4	4	2	1
***E. faecalis*** **C9884**	*erm*(B), *lnu*(B), *lsa*(A), *lsa*(E), *aac*(6′)-*aph*(2″), *aph*(3″)-III, *ant*(6)-Ia, *str*, *tet*(M), *tet*(L), *optrA*, *fexA*, *dfrG*	>256	256	128	64	64	16	1
***E. faecalis*** **TC-C9884**	***optrA*** **, *fexA***	1	2	0.125	32	64	8	1
***E. faecalis*** **C9901**	*erm*(B), *lnu*(B), *lsa*(A), *lsa*(E), *aph*(3″)-III, *ant*(6)-Ia, *tet*(M), *tet*(L), *optrA*, *fexA*	>256	>256	>256	32	64	16	1
***E. faecalis*** **TC-C9901**	***optrA*** **, *fexA***	1	2	0.125	32	64	4	1
***E. faecalis*** **C9951**	*erm*(A)-like, *erm*(B), *lnu*(B), *lsa*(A), *lsa*(E), *aac*(6′)-*aph*(2″), *aph*(3″)-III, *ant*(6)-Ia, *tet*(M), *tet*(L), *tet*(O), *optrA*, *fexA*, *dfrG*	>256	>256	128	64	64	8	1
***E. faecalis*** **TC-C9951**	***erm*** **(A)-like, *tet*(L), *tet*(O), *optrA*, *fexA***	256	>256	128	64	64	4	1
***E. faecalis*** **C9952**	*erm*(A)-like, *erm*(B), *lnu*(B), *lsa*(A), *lsa*(E), *aac*(6′)-*aph*(2″), *aph*(3″)-III, *ant*(6)-Ia, *tet*(M), *tet*(L), *optrA*, *fexA*	256	>256	>256	32	64	8	1
***E. faecalis*** **TC-C9952**	***erm*** **(A)-like, *optrA*, *fexA***	128	>256	0.125	32	64	4	1
***E. faecalis*** **C9736**	*erm*(B), *lnu*(B), *lsa*(A), *lsa*(E), *aac*(6′)-*aph*(2″), *aph*(3″)-III, *ant*(6)-Ia, *str*, *tet*(M), *tet*(L), *optrA*, *fexA*, *dfrG*	>256	128	256	128	32	16	1
***E. faecalis*** **TC-C9736**	***optrA*** **, *fexA***	1	2	0.125	64	32	8	1
***E. faecalis*** **C9737**	*erm*(B), *lnu*(B), *lsa*(A), *lsa*(E), *aph*(3″)-III, *ant*(6)-Ia, *tet*(M), *optrA*, *fexA*, *cat*_pC221_	>256	128	128	32	64	16	1
***E. faecalis*** **TC-C9737**	***optrA*** **, *fexA***	1	2	0.125	32	32	8	1
***E. faecalis*** **X523**	*lsa*(A), *lsa*(E), *tet*(M), *tet*(L), *optrA*, *fexA, dfrG*	128	>256	128	32	64	8	1
***E. faecalis*** **TC-X523**	***optrA*** **, *fexA***	1	2	0.125	32	64	8	1
***E. faecalis*** **X524**	*erm*(B), *lsa*(A), *lsa*(E), *aac*(6′)-*aph*(2″), *aph*(3″)-III, *ant*(6)-Ia, *tet*(M), *tet*(L), *optrA*, *fexA*, *dfrG*	256	256	256	32	64	8	1
***E. faecalis*** **TC-X524**	***optrA*** **, *fexA***	1	2	0.125	32	64	8	1
***E. faecalis*** **X525**	*erm*(B), *lnu*(B), *lsa*(A), *lsa*(E), *aac*(6′)-*aph*(2″), *aph*(3″)-III, *ant*(6)-Ia, *str*, *tet*(M), *tet*(L), *optrA*, *fexA*, *catA*, *dfrG*	>256	128	128	64	64	8	1
***E. faecalis*** **TC-X525**	***optrA*** **, *fexA***	1	2	0.125	32	32	8	1
***E. faecalis*** **X526**	*erm*(B), *lnu*(B), *lsa*(A), *lsa*(E), *aph*(3″)-III, *ant*(6)-Ia, *tet*(M), *optrA*, *fexA*, *cat*_pC221_	>256	128	128	32	64	4	1
***E. faecalis*** **TC-X526**	***optrA*** **, *fexA***	1	2	0.125	32	64	4	1
***E. faecalis*** **X527**	*erm*(B), *lsa*(A), *aac*(6′)-*aph*(2″), *aph*(3″)-III, *ant*(6)-Ia, *tet*(M), *optrA*, *fexA*	>256	256	128	32	64	16	1
***E. faecalis*** **TC-X527**	***optrA*** **, *fexA***	1	2	0.125	32	64	8	1
***E. faecalis*** **X528**	*erm*(B)*, lsa*(A), *aph*(3″)-III, *ant*(6)-Ia, *tet*(M), *tet*(L), *cfr*(D), *optrA*, *fexA, dfrG*	>256	>256	>256	32	64	16	1
***E. faecalis*** **TC-X528**	***optrA*** **, *fexA***	1	2	0.125	32	64	8	1

^1^ ERY, erythromycin; CLI, clindamycin; TET, tetracycline; CHL, chloramphenicol; FFC, florfenicol; LZD, linezolid; TDZ, tedizolid; the genotypes of the transconjugants are displayed in bold.

## Supplementary Materials

The following are available online at https://www.mdpi.com/2076-2607/8/8/1155/s1. Table S1: Primer pairs used for the molecular typing, and the detection of antimicrobial resistance and virulence genes. Figure S1: Location of the six Spanish hospitals that took part in this study. Figure S2: Phylogenetic relatedness of the 13 *E. faecalis* isolates based on single nucleotide polymorphism (SNP) analysis performed using CSI Phylogeny. Figure S3: Pulsed field gel electrophoresis (PFGE) of whole-cell DNA of *E. faecium* isolates after digestion with the enzyme *Sma*I.
