# Supplementary material for: Mechanisms of Linezolid Resistance Among Enterococci of Clinical Origin in Spain—Detection of optrA- and cfr(D)-Carrying E. faecalis
Source: Microorganisms. 2020 Jul 30;8(8):1155. doi: 10.3390/microorganisms8081155 (PMC7464793; doi:10.3390/microorganisms8081155)
Supplement: Supplementary file 1 [file microorganisms-08-01155-s001.pdf]

# Mechanisms of Linezolid Resistance Among Enterococci of Clinical Origin in Spain—Detection of *optrA*- and *cfr*(D)-Carrying *E. faecalis*

**Figure S1.** Location of the six Spanish hospitals that took part in this study.

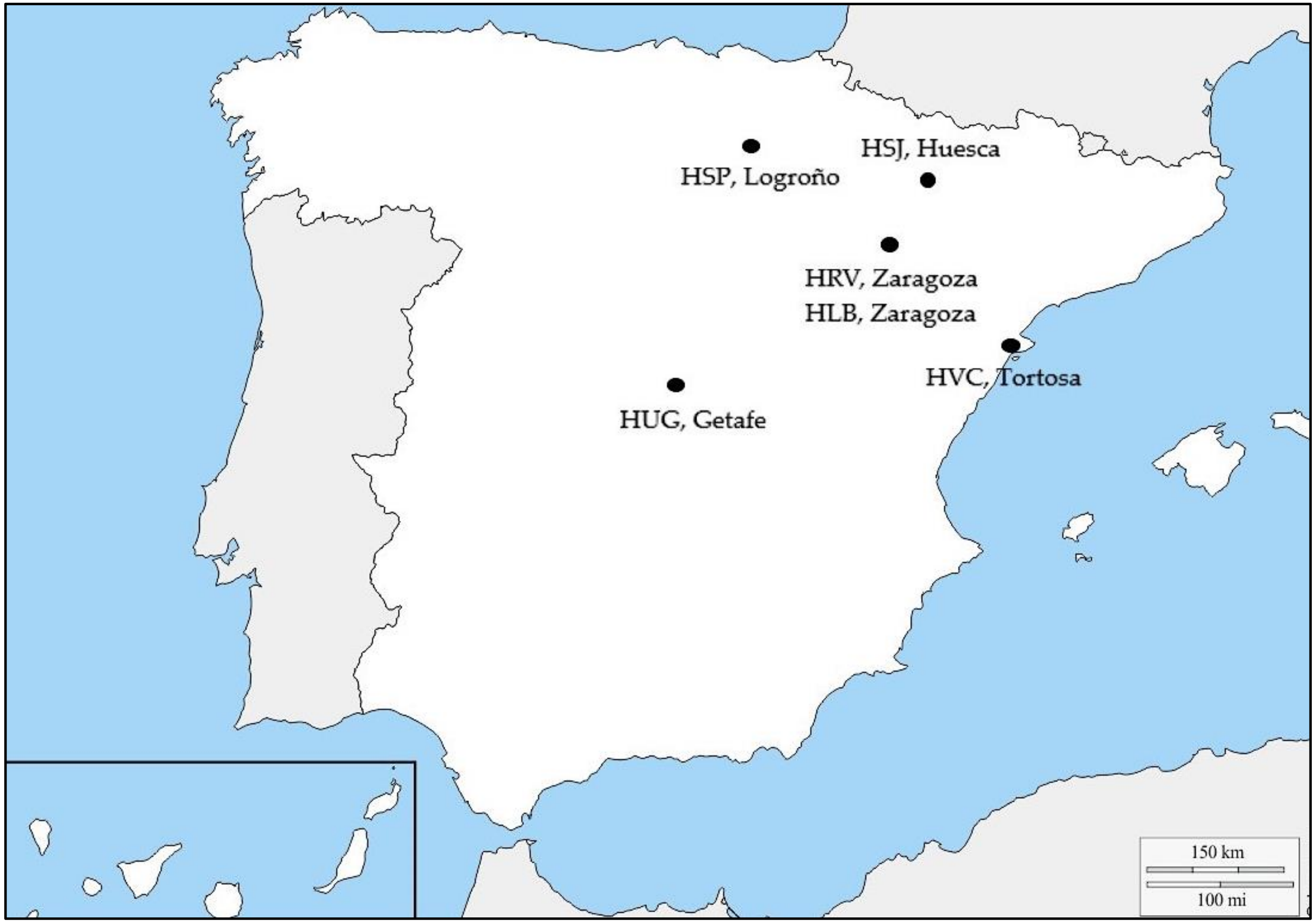


HSJ, Hospital San Jorge; HUG, Hospital Universitario de Getafe; HSP, Hospital San Pedro; HVC, Hospital Verge de la Cinta; HLB, Hospital Lozano Blesa; HRV, Hospital Royo Villanova.

**Table S1**. Primer pairs used for the molecular typing, and the detection of antimicrobial resistance and virulence genes.

| Target gene | Primer Sequence (5′-3′) | Amplicon size (bp) | Reference |
|---|---|---|---|
| **Multilocus sequence typing *E. faecium*** | | | |
| *adk* | F: TATGAACCTCATTTTAATGGG | 437 | [1] |
| | R: GTTGACTGCCAAACGATTTT | | |
| *atpA* | F: TTCAAATGGCTCATACGG | 556 | |
| | R: AGTTCACGATAAGCAACAGC | | |
| *ddl* | F: GAGACATTGAATATGCCTTAT | 465 | |
| | R: AAAAAGAAATCGCACCG | | |
| *gdh* | F: GGCGCACTAAAAGATATGGT | 530 | |
| | R: CCAAGATTGGGCAACTTCGTCCCA | | |
| *pstS* | F: TTGAGCCAAGTCGAAGCTGGA | 583 | |
| | R: CGTGATCACGTTCTACTTCC | | |
| *gyd* | F: CAAACTGCTTAGCTCCAAGGC | 395 | |
| | R: CATTTCGTTGTCATACCAAGC | | |
| *purK* | F: GCAGATTGGCACATTGAAAGT | 492 | |
| | R:TACATAAATCCCGCCTGTTTY | | |
| **Multilocus sequence typing *E. faecalis*** | | | |
| *aroE* | F: TGGAAAACTTTACGGAGACAGC | 459 | [2] |
| | R: GTCCTGTCCATTGTTCAAAAGC | | |
| *gki* | F: GATTTTGTGGGAATTGGTATGG | 438 | |
| | R: ACCATTAAAGCAAAATGATCGC | | |
| *pstS* | F: CGGAACAGGACTTTCGC | 583 | |
| | R: ATTTACATCACGTTCTACTTGC | | |

| | | | |
|---|---|---|---|
| *xpt* | F: AAAATGATGGCCGTGTATTAGG<br>R: AACGTCACCGTTCCTTCACTTA | 456 | |
| *yqil* | F: CAGCTTAAGTCAAGTAAGTGCCG<br>R: GAATATCCCTTCTGCTTGTGCT | 436 | |
| *gdh* | F: GGCGCACTAAAAGATATGGT<br>R: CCAAGATTGGGCAACTTCGTCCCA | 530 | |
| *gyd* | F: CAAACTGCTTAGCTCCAAGGC<br>R: CATTTCGTTGTCATACCAAGC | 395 | |
| **Resistance genes** | | | |
| *optrA* | F: AGGTGGTCAGCGAACTAA<br>R: ATCAACTGTTCCCATTCA | 1395 | [3] |
| *poxtA* | F: TCAATGCAGAGCAGGAAGCA<br>R: GGTGGATTTACCGACACCGT | 791 | [4] |
| *cfr* | F: TGAAGTATAAAGCAGGTTGGGAGTCA<br>R: ACCATATAATTGACCACAAGCAGC | 746 | [5] |
| *cfr*(B) | F: TGAGCATATACGAGTAACCTCAAGA<br>R: CGCAAGCAGCGTCTATATCA | 293 | [6] |
| *cfr*(D) | F: AGAAGTCGCAACAAGTGAGGA<br>R: GCAACTGCATGAGTCAAAGAA | 595 | This study[1] |
| *fexA* | F: GTACTTGTAGGTGCAATTACGGCTGA<br>R: CGCATCTGAGTAGGACATAGCGTC | 1272 | [7] |
| *fexB* | F: TTCCCACTATTGGTGAAAGGAT<br>R: GCAATTCCCTTTTATGGACGTT | 816 | [8] |

| | | | |
|---|---|---|---|
| $cat_{pC194}$ | F: CGACTTTTAGTATAACCACAGA<br>R: GCCAGTCATTAGGCCTAT | 570 | [9] |
| $cat_{pC221}$ | F: ATTTATGCAATTATGGAAGTTG<br>R: TGAAGCATGGTAACCATCAC | 434 | |
| $cat_{pC223}$ | F: GAATCAAATGCTAGTTTTAACTC<br>R: ACATGGTAACCATCACATAC | 283 | |
| *catA* | F: GGATATGAAATTTATCCCTC<br>R: CAATCATCTACCCTATGAAT | 505 | [10] |
| *erm*(A) | F: TCTAAAAAGCATGTAAAAGAA<br>R: CTTCGATAGTTTATTAATATTAG | 645 | [11] |
| *erm*(B) | F: GAAAAGTACTCAACCAAATA<br>R: AGTAACGGTACTTAAATTGTTTA | 639 | |
| *erm*(C) | F: TCAAAACATAATATAGATAAA<br>R: GCAAATATTGTTTAAATCGTCAAT | 642 | |
| *msr*(C) | F: CTAGATGGGTTGTTGGCTCGT<br>R: AGCTTTGGCAACCAGCTAGAA | 361 | [12] |
| *lnu*(B) | F: CCTACCTATTGTTTGTGGAA<br>R: ATAACGTTACTCTCCTATTC | 944 | [13] |
| *lsa*(B) | F: TGCCGAAGCCATGTACCGTCC<br>R: CGGTTAGACCAACCAGCCGAACG | 396 | [14] |
| *lsa*(E) | F: CGGCTATAGAACCGTTTGTTTT<br>R: AGTTATTGTGGCAACTCAAAATC | 1819 | [15] |
| *aac*(6')-Ii | F: GCGGTAGCAGCGGTAGACCAAG<br>R: GCATTTGGTAAGACACCTACG | 323 | [16] |

| | | | |
|---|---|---|---|
| *aac*(6')-Ie-*aph*(2'')-Ia | F: CCAAGAGCAATAAGGGCATA<br>R: CACTATCATAACCACTACCG | 220 | [17] |
| *aph*(3')-III | F: GCCGATGTGGATTGCGAAAA<br>R: GCTTGATCCCCAGTAAGTCA | 282 | |
| *ant*(6)-Ia | F: ACTGGCTTAATCAATTTGGG<br>R: GCCTTTCCGCCACCTCACCG | 597 | [18] |
| *str* | F: TATTGCTCTCGAGGGTTC<br>R: CTTTCTATATCCATTCATCTC | 646 | [9] |
| *ant*(4')-Ia | F: GCAAGGACCGACAACATTTC<br>R: TGGCACAGATGGTCATAACC | 165 | [17] |
| *tet*(K) | F: TTAGGTGAAGGGTTAGGTCC<br>R: GCAAACTCATTCCAGAAGCA | 697 | [10] |
| *tet*(L) | F: CATTTGGTCTTATTGGATCG<br>R: ATTACACTTCCGATTTCGG | 456 | |
| *tet*(M) | F: GTTAAATAGTGTTCTTGGAG<br>R: CTAAGATATGGCTCTAACAA | 576 | |
| *tet*(O) | F: ACGGARAGTTTATTGTATACC<br>R: TGGCGTATCTATAATGTTGAC | 171 | [19] |
| *dfrA* | F: CCTTGGCACTTACCAAATG<br>R: CTGAAGATTCGACTTCCC | 374 | [9] |
| *dfrD* | F: TTCTTTAATTGTTGCGATGG<br>R: TTAACGAATTCTCTCATATATATG | 582 | |
| *dfrG* | F: TCGGAAGAGCCTTACCTGACAGAA<br>R: CCCTTTTTGGGCAAATACCTCATTCCA | 323 | [14] |

| | | | |
|---|---|---|---|
| *dfrK* | F: GAGAATCCCAGAGGATTGGG<br>R: CAAGAAGCTTTTCGCTCATAAA | 423 | |
| **Mutations** | | | |
| 23S ARN | F: GCGGTCGCCTCCTAAAAG<br>R: ATCCCGGTCCTCTCGTACT | 420 | [20] |
| *rplC* (L3) | F: ATGACCAAAGGAATCTTAGGG<br>R: CACAGCTGATTTGATWGTGATT | 618 | [21] |
| *rplD* (L4) | F: GCCGAATGTAGCATTATTCAA<br>R: CAAGCACCTCCTCAATTTGAGT | 617 | |
| *rplV* (L22) (*E. faecium*) | F: GGACATGCTGCTGACGATA<br>R: ACCATTTAGCATCCCAGTCG | 486 | [6] |
| *rplV* (L22) (*E. faecalis*) | F: GCCACGTTGCTGACGATAA<br>R: ACCCACTGATTGTCCCTCCT | 476 | |
| *gyrA* | F: CGGGATGAACGAATTGGGTGTGA<br>R: AATTTTACTCATACGTGCTTCGG | 240 | [22] |
| *parC* | F: TTCCCGTGCATTTCGATCAGTACTTC<br>R: CGTATGACAAAGGATTCGGTAAATC | 151 | |
| *pbp5* | F : CGGGATCTCACAAGAAGAT<br>R : TTATTGATAATTTTGGTT | 861 | [23] |
| | F : AAAAATCGAACAGGCGCTTA<br>R : TTGTGAGATCCCGTTTGTCA | 1140 | [24] |
| **Genetic environment of the *optrA* gene** | | | |
| *optrA-fexA* | F: TTAGTTCGCTGACCACCT<br>R: GACGCTATGTCCTACTCAGATGCG | 1348 | This study[2] |

| | | | |
|---|---|---|---|
| **Virulence factors** | | | |
| *esp* | F: TTGCTAATGCTAGTCCACGACC<br>R: GCGTCAACACTTGCATTGCCGAA | 933 | [25] |
| *hyl* | F: GAGTAGAGGAATATCTTAGC<br>R: AGGCTCCAATTCTGT | 661 | [26] |
| *ace* | F: GGAATGACCGAGAACGATGGC<br>R: GCTTGATGTTGGCCTGCTTCCG | 616 | [27] |
| *agg* | F: AAGAAAAAGAAGTAGACCAAC<br>R: AAACGGCAAGACAAGTAAATA | 1553 | [25] |
| *gelE* | F: AGTTCATGTCTATTTTCTTCAC<br>R: CTTCATTATTTACACGTTTG | 402 | |

[1]The *cfr*(D) PCR was performed using the following conditions: 94°C for 7 min, followed by 30 cycles of 1 min at 94°C, 1 min at 60°C, and 1 min at 72°C, with a final extension at 72°C for 10 min.
[2]The *optrA*-*fexA* PCR was performed using the following conditions: 94°C for 7 min, followed by 30 cycles of 1 min at 94°C, 1 min at 60°C, and 2 min at 72°C, with a final extension at 72°C for 10 min.

**Figure S2.** Phylogenetic relatedness of the 13 *E. faecalis* isolates based on single nucleotide polymorphism (SNP) analysis performed using CSI Phylogeny.

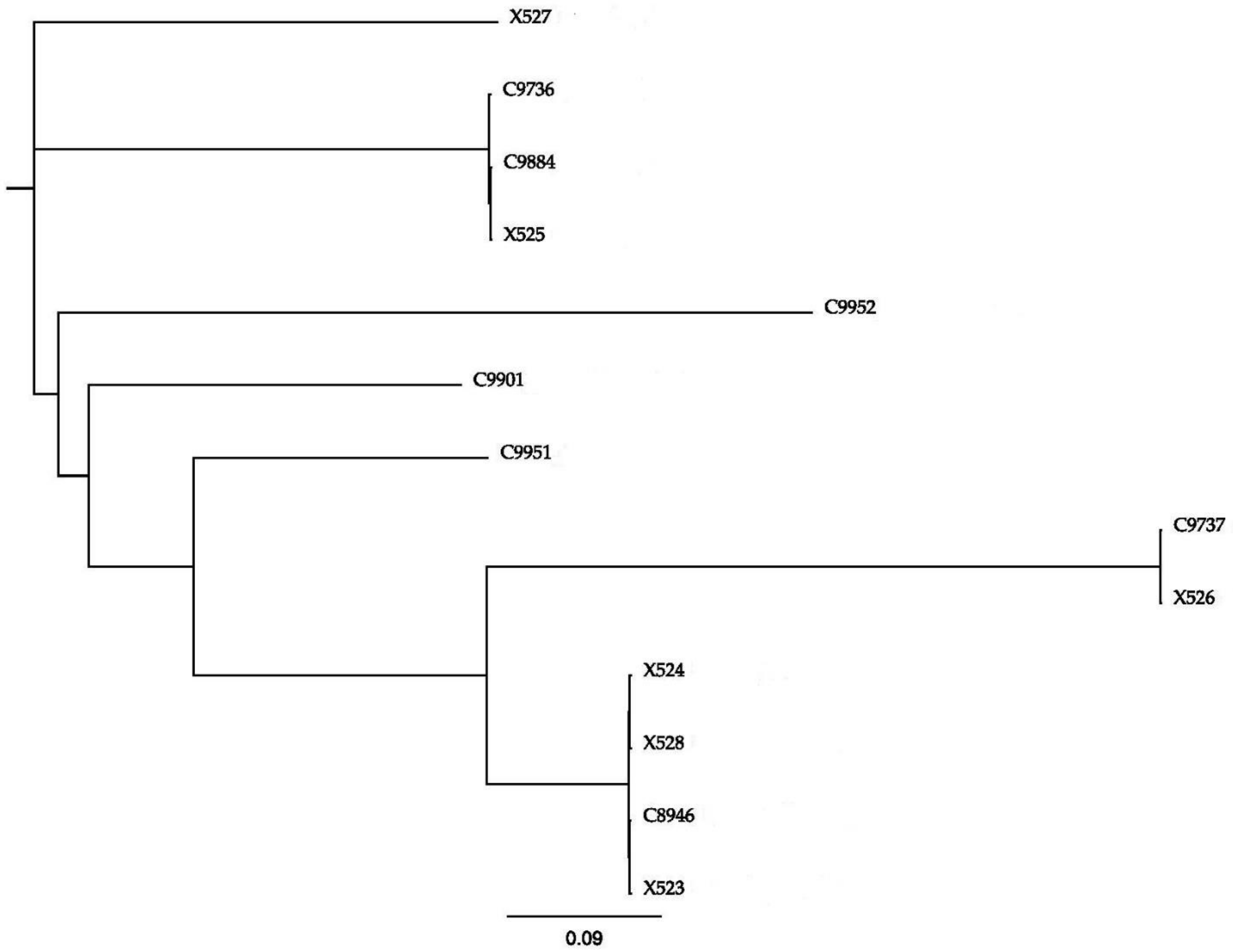


The scale bar indicates the number of substitutions per site. The reference *E. faecalis* ATCC® 29212 was not included in final phylogeny.

**Figure S3.** Pulsed field gel electrophoresis (PFGE) of whole-cell DNA of *E. faecium* isolates after digestion with the enzyme *Sma*I.

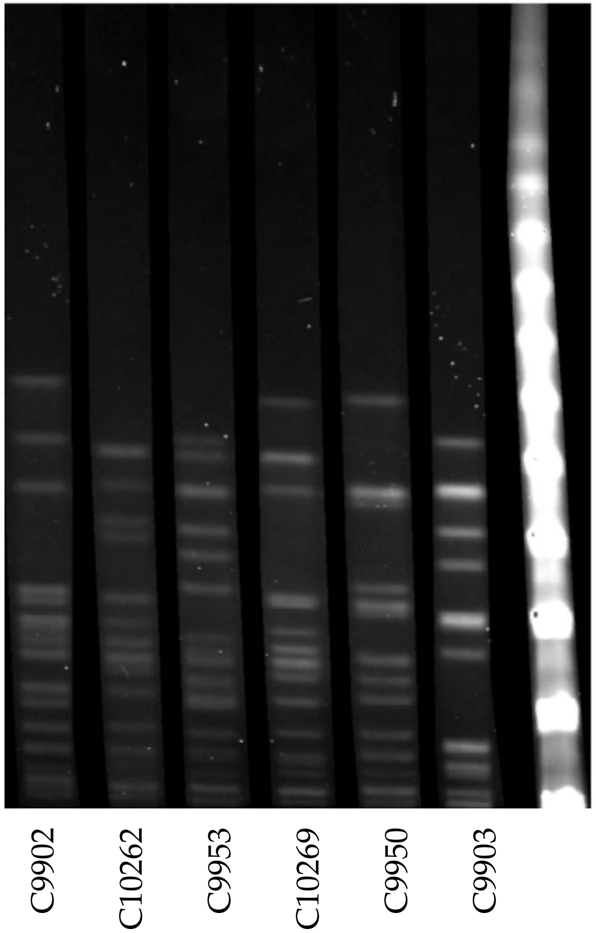

## References


1. Homan, W.L.; Tribe, D.; Poznanski, S.; Li, M.; Hogg, G.; Spalburg, E.; Van Embden, J.D.; Willems, R.J. Multilocus sequence typing scheme for *Enterococcus faecium*. *J. Clin. Microbiol.* **2002**, *40*, 1963-1971. https://doi.org/10.1128/jcm.40.6.1963-1971.2002.
2. Ruiz-Garbajosa, P.; Bonten, M.J.; Robinson, D.A.; Top, J.; Nallapareddy, S.R.; Torres, C.; Coque, T.M.; Cantón, R.; Baquero, F.; Murray, B.E.; et al. Multilocus sequence typing scheme for *Enterococcus faecalis* reveals hospital-adapted genetic complexes in a background of high rates of recombination. *J. Clin. Microbiol.* **2006**, *44*, 2220-2228. https://doi.org/10.1128/JCM.02596-05.
3. Wang, Y.; Lv, Y.; Cai, J.; Schwarz, S.; Cui, L.; Hu, Z.; Zhang, R.; Li, J.; Zhao, Q.; He, T.; et al. A novel gene, *optrA*, that confers transferable resistance to oxazolidinones and phenicols and its presence in *Enterococcus faecalis* and *Enterococcus faecium* of human and animal origin. *J. Antimicrob. Chemother.* **2015**, *70*, 2182-2190. https://doi.org/10.1093/jac/dkv116.
4. Ruiz-Ripa, L.; Feßler, A.T.; Hanke, D.; Sanz, S.; Olarte, C.; Mama, O.M.; Eichhorn, I.; Schwarz, S.; Torres, C. Coagulase-negative staphylococci carrying *cfr* and PVL genes, and MRSA/MSSA-CC398 in the swine farm environment. *Vet. Microbiol.* **2020**, *243*, 108631. https://doi.org/10.1016/j.vetmic.2020.108631.
5. Kehrenberg, C.; Schwarz, S. Distribution of florfenicol resistance genes *fexA* and *cfr* among chloramphenicol-resistant *Staphylococcus* isolates. *Antimicrob. Agents Chemother.* **2006**, *50*, 1156-1163. https://doi.org/10.1128/AAC.50.4.1156-1163.2006.
6. Lee, S.M.; Huh, H.J.; Song, D.J.; Shim, H.J.; Park, K.S.; Kang, C.I.; Ki, C.S.; Lee, N.Y. Resistance mechanisms of linezolid-nonsusceptible enterococci in Korea: low rate of 23S rRNA mutations in *Enterococcus faecium*. *J. Med. Microbiol.* **2017**, *66*, 1730-1735. https://doi.org/10.1099/jmm.0.000637.
7. Kehrenberg, C.; Schwarz, S. Florfenicol-chloramphenicol exporter gene *fexA* is part of the novel transposon Tn*558*. *Antimicrob. Agents Chemother.* **2005**, *49*, 813-815. https://doi.org/10.1128/AAC.49.2.813-815.2005.
8. Liu, H.; Wang, Y.; Wu, C.; Schwarz, S.; Shen, Z.; Jeon, B.; Ding, S.; Zhang, Q.; Shen, J.A. A novel phenicol exporter gene, *fexB*, found in enterococci of animal origin. J. *Antimicrob. Chemother.* **2012**, *67*, 322-325. https://doi.org/10.1093/jac/dkr481.
9. Schnellmann, C.; Gerber, V.; Rossano, A.; Jaquier, V.; Panchaud, Y.; Doherr, M.G.; Thomann, A.; Straub, R.; Perreten, V. Presence of new *mecA* and *mph*(C) variants conferring antibiotic resistance in *Staphylococcus* spp. isolated from the skin of horses before and after clinic admission. *J. Clin. Microbiol.* **2006**, *44*, 4444-4454. https://doi.org/10.1128/JCM.00868-06.
10. Aarestrup, F.M.; Agerso, Y.; Gerner-Smidt, P.; Madsen, M.; Jensen, L.B. Comparison of antimicrobial resistance phenotypes and resistance genes in *Enterococcus faecalis* and *Enterococcus faecium* from humans in the community, broilers, and pigs in Denmark. *Diagn. Microbiol. Infect. Dis.* **2000**, *37*, 127-137. https://doi.org/10.1016/s0732-8893(00)00130-9.
11. Sutcliffe, J.; Grebe, T.; Tait-Kamradt, A.; Wondrack, L. Detection of erythromycin-resistant determinants by PCR. *Antimicrob. Agents Chemother.* **1996**, *40*, 2562-2566.
12. Ruiz-Ripa, L.; Feßler, A.T.; Hanke, D.; Sanz, S.; Olarte, C.; Eichhorn, I.; Schwarz, S.; Torres, C. Detection of *poxtA*- and *optrA*-carrying *E. fae*cium isolates in air samples of a Spanish swine farm. *J. Glob. Antimicrob. Resist.* **2019**, pii, S2213-7165(19)30326-1. https://doi.org/10.1016/j.jgar.2019.12.012.
13. Bozdogan, B.; Berrezouga, L.; Kuo, M.S.; Yurek, D.A.; Farley, K.A.; Stockman, B.J.; Leclercq, R. A new resistance gene, *linB*, conferring resistance to lincosamides by nucleotidylation in *Enterococcus faecium* HM1025. *Antimicrob. Agents Chemother*. **1999**, *43*, 925-929.

14. Gómez-Sanz, E.; Torres, C.; Lozano, C.; Fernández-Pérez, R.; Aspiroz, C.; Ruiz-Larrea, F.; Zarazaga, M. Detection, molecular characterization, and clonal diversity of methicillin-resistant *Staphylococcus aureus* CC398 and CC97 in Spanish slaughter pigs of different age groups. *Foodborne Pathog. Dis.* **2010**, *27*, 1269-1277. https://doi.org/10.1089/fpd.2010.0610.
15. Wendlandt, S.; Lozano, C.; Kadlec, K.; Gómez-Sanz, E.; Zarazaga, M.; Torres, C.; Schwarz, S. The enterococcal ABC transporter gene *lsa*(E) confers combined resistance to lincosamides, pleuromutilins and streptogramin A antibiotics in methicillin-susceptible and methicillin-resistant *Staphylococcus aureus*. *J. Antimicrob. Chemother.* **2013**, *68*, 473-475. https://doi.org/10.1093/jac/dks398.
16. Costa, Y.; Galimand, M.; Leclercq, R.; Duval, J.; Courvalin, P. Characterization of the chromosomal *aac*(6')-Ii gene specific for *Enterococcus faecium*. *Antimicrob. Agents Chemother.* **1993**, *37*, 1896-1903. https://doi.org/10.1128/aac.37.9.1896.
17. van de Klundert, J.A.M.; Vliegenthart, J.S. PCR detection of genes coding for aminoglycoside-modifying enzymes In *Diagnostic Molecular Microbiology. Principles and applications*; Persing, D.H., Smith, T.F., Tenover, F.C., Wite, T.J. Eds.; ASM, Washington, USA, 1993.
18. Clark, N.C.; Olsvik, O.; Swenson, J.M.; Spiegel, C.A.; Tenover, F.C. Detection of a streptomycin/spectinomycin adenylyltransferase gene (*aadA*) in *Enterococcus faecalis*. *Antimicrob. Agents Chemother.* **1999**, *43*, 157-160.
19. Aminov, R.I.; Garrigues-Jeanjean, N.; Mackie, R.I. Molecular ecology of tetracycline resistance: development and validation of primers for detection of tetracycline resistance genes encoding ribosomal protection proteins. *Appl. Environ. Microbiol.* **2001**, *67*, 22-32. https://doi.org/10.1128/AEM.67.1.22-32.2001.
20. Dibo, I.; Pillai, S.K.; Gold, H.S.; Baer, M.R.; Wetzler, M.; Slack, J.L.; Hazamy, P.A.; Ball, D.; Hsiao, C.B.; McCarthy, P.L.; et al. Linezolid-resistant *Enterococcus faecalis* isolated from a cord blood transplant recipient. *J. Clin. Microbiol.* **2004**, *42*, 1843-1845. https://doi.org/10.1128/jcm.42.4.1843-1845.2004.
21. Diaz, L.; Kiratisin, P.; Mendes, R.E.; Panesso, D.; Singh, K.V.; Arias, C.A. Transferable plasmid-mediated resistance to linezolid due to *cfr* in a human clinical isolate of *Enterococcus faecalis*. *Antimicrob. Agents Chemother.* **2012**, *56*, 3917-3922. https://doi.org/10.1128/AAC.00419-12.
22. Leavis, H.L.; Willems, R.J.; Top, J.; Bonten, M.J. High-level ciprofloxacin resistance from point mutations in *gyrA* and *parC* confined to global hospital-adapted clonal lineage CC17 of *Enterococcus faecium*. *J. Clin. Microbiol.* **2006**, *44*, 1059-1064. https://doi.org/10.1128/JCM.44.3.1059-1064.2006.
23. Hsieh, S.; Hsu, L.; Hsu, W.; Chen, C.; Chen, H.; Liao, C. Importance of amino acid alterations and expression of penicillin-binding protein 5 to ampicillin resistance of *Enterococcus faecium* in Taiwan. *Int. J. Antimicrob. Agents*. **2006**, *28*, 514-519. https://doi.org/10.1016/j.ijantimicag.2006.07.027.
24. Jureen, R.; Top, J.; Mohn, S.C.; Harthug, S.; Langeland, N.; Willems, R.J.L. Molecular characterization of ampicillin-resistant *Enterococcus faecium* isolates from hospitalized patients in Norway. *J. Clin. Microbiol.* **2003**, *41*, 2330-2336. https://doi.org/1128/jcm.41.6.2330-2336.2003.
25. Eaton, T.J.; Gasson, M.J. Molecular screening of *Enterococcus* virulence determinants and potential for genetic exchange between food and medical isolates. *Appl. Environ. Microbiol.* **2001**, *67*, 1628-1635. https://doi.org/10.1128/AEM.67.4.1628-1635.2001.
26. Klare, I.; Konstabel, C.; Mueller-Bertling, S.; Werner, G.; Strommenger, B.; Kettlitz, C.; Borgmann, S.; Schulte, B.; Jonas, D.; Serr, A.; et al. Spread of ampicillin/vancomycin-resistant *Enterococcus faecium* of the epidemic-virulent clonal complex-17 carrying the genes *esp* and *hyl* in German hospitals. *Eur. J. Clin. Microbiol. Infect. Dis.* **2005**, *24*, 815-825. https://doi.org/10.1007/s10096-005-0056-0.
27. Creti, R.; Imperi, M.; Bertuccini, L.; Fabretti, F.; Orefici, G.; Di Rosa, R.; Baldassarri, L. Survey for virulence determinants among *Enterococcus faecalis* isolated from different sources. *J. Med. Microbiol.* **2004**, *53*, 13-20. https://doi.org/10.1099/jmm.0.05353-0.
